# Juvenile idiopathic recurrent parotitis (JIRP) treated with short course steroids, a case series study and one decade follow up for potential autoimmune disorder

**DOI:** 10.1186/s12969-023-00946-0

**Published:** 2024-01-04

**Authors:** Farhad Salehzadeh, Rasol Molatefi, Ali Mardi, Negin Nahanmoghaddam

**Affiliations:** 1 Pediatric Rheumatology, Pediatric Department, Bouali Children’s Hospital, Ardabil University of Medical Sciences (ARUMS), Ardabil, Iran; 2 Pediatric Allergy and Immunology, Pediatric Department, Bouali Children’s Hospital, Ardabil University of Medical Sciences (ARUMS), Ardabil, Iran; 3 Pediatric Gastroenterology, Pediatric Department, Bouali Children’s Hospital, Ardabil University of Medical Sciences (ARUMS), Ardabil, Iran; 4 Pediatric Infectious Disease, Pediatric Department, Bouali Children’s Hospital, Ardabil University of Medical Sciences (ARUMS), Ardabil, Iran

**Keywords:** Parotitis, Recurrent parotitis, Juvenile, Prednisolone

## Abstract

**Background:**

Juvenile idiopathic recurrent parotitis (JIRP) in children is a condition characterized with recurrent episodes of idiopathic parotid gland inflammation. Since there are no definitive guidelines for diagnosis and management of this condition, we present a consecutive case series of patients with more than one decade follow up and their dramatic response to short course treatment by prednisolone.

**Methods:**

We conducted this study by retrospectively reviewed medical charts of children who were diagnosed with JIRP, from 1 January 2002 to 29 February 2023. We performed usual serological tests to exclude some possible background. We administered short course prednisolone on first day of episode as divided dosage (0.5 mg /kg).

**Results:**

In this case series of 10 patients (70%) were male, median age of onset was 5 years, duration of episodes 5 days, and the mean course of disease were 3.8 years. The average follows up of patients was near 10 years. In comparison with their natural course of disease all patients showed a dramatic response to treatment on the first day of administration of prednisolone (P Value 0.005). For ten years follow up there was not any additional accompanying autoimmune disorder.

**Conclusion:**

Short course prednisolone on first day of each episode and its dramatic and meaningful response in our patients, introduce a new, effective, fast, and inexpensive regimen of therapy in patients with JIRP.

**Supplementary Information:**

The online version contains supplementary material available at 10.1186/s12969-023-00946-0.

## Introduction

Juvenile idiopathic recurrent parotitis (JIRP) in children is a condition characterized with recurrent episodes of idiopathic non-obstructive, non-suppurative parotid gland inflammation during childhood. It usually manifests with pain, swelling and fever of less than 1 week duration and are separated by asymptomatic periods [1, 2].

Although the exact incidence is not clear, it is known as uncommon clinical condition [3, 4, and 5]. JIRP was the second most frequent disease of the parotid gland in children before the program of mumps vaccine [6, 7].

It presents with a male preponderance, mostly unilateral and usually occur between 3 and 6 years, and tends to resolve during puberty in most patients [1, 8].

The diagnosis of JIRP is mainly based on history and physical examination with ≥ 2 attacks of acute parotitis [3]. Preliminary diagnosis is frequently confirmed by ultrasonography (US) after excluding other possible causes, such as ductal malformation, sialoliths, acute infectious parotitis, tumors and systemic allergic or autoimmune disorders [9–11].

Its etiology is not fully understood. It is believed that multifactorial parameters are involved in pathophysiology of JIRP. One possible explanation is reduced flow or modified content of saliva which induces stasis, and recruitment of inflammation [4, 9]. Some researchers suggest structural anomaly of the ducts as the main trigger for secondary inflammation [4]. Furthermore, viral and bacterial infections, malocclusion, autoimmune diseases (e.g. Sjogren and sarcoidosis), and immune disorders such as selective Ig A deficiency have been reported as accompanying diseases [3, 5, 10, 12–17].

The treatment of JIRP generally consists of relief of acute symptoms. First step measures are maintenance of good oral hygiene, adequate hydration, warm massage to the parotid gland, and use of analgesics [11]. The administration of antibiotics during attacks is controversial [4, 18, 19]. More recently, increasing evidence indicate success of sialendoscopy in the diagnosis and treatment of JIRP [20].

Since there are no definitive guidelines for diagnosis and management of this condition and patients may be undergo unnecessary and potentially invasive procedures, we determined to present a consecutive case series of patients with more than one decade follow up and we evaluated their response to short course treatments by prednisolone, additionally we reviewed current modality of diagnosis and therapy.

## Methods

We conducted this study to evaluate our two decades JIRP patients, and we retrospectively reviewed medical charts of children who were diagnosed with JIRP at Ardabil University of medical Sciences (ARUMS) at Bouali Children’s Hospital between 2002 and 2023. All patients had received MMR and Hepatitis B vaccine.

Recurrent parotitis of childhood was defined as two or more episodes of parotid swelling, often with pain, fever, and without evidence of suppuration.

Patients were identified by reviewing the medical records with the diagnosis of parotitis or sialadenitis over a 21-year period from 1 January 2002 to 29 February 2023. Parotid involvement was confirmed in all cases by compatible clinical and sonographic findings.

We collected data on basic clinical variables, such as the age at onset, sex, location, duration, course of disease, and we checked serological tests primary and if needed serially to exclude some possible infectious and rheumatological disease such as:

Complete blood count, blood chemistry panel, CRP and ESR.

Rheumatoid factor Immunoglobulin levels C3, C4.

Tissue transglutaminase (tTG) IgA levels (and IgG in case of IgA deficiency).

Autoantibodies: antinuclear (ANA) and anti-Ro/SSA, anti-La (SSB) antibodies and rheumatoid factor.

Viral/Bacterial detection tests (CMV, EBV, and HIV).

Mantoux test Parotid gland ultrasound and in some cases sweat chloride test and Chest radiograph.

In addition of massage, analgesic, and warmness we administered all patients short course prednisolone on first day of each episode as divided dosage 10–15 mg /po (0.5 mg/kg) dependent of patient’s age and weight. Response to therapy was defined by the resolving of the swelling, pain, and fever.

Patients had received antibiotics at some stage to treat their parotitis, particularly at their first episodes of disease.

The statistical analysis of the data, with calculation of percentages, mean values and paired t test (Wilcoxon) in comparison before and after treatment was performed by SPSS Version 26. The study was approved by the Scientific and Ethics Research Committee of the faculty (file no. IR.ARUMS.REC.1393.04760). Informed consent was obtained from all parents of participants included in the study.

## Results

In this case series of 10 patients 70% were male, median age of onset was 5 years, duration of episodes 5 days, and the mean course of the disease were 3.8 years (Table 1).

**Table 1:** Characteristic findings of patients

NO	SEX	Age/onset/yr	Parotitis	Interval/ mo.	Duration /Day	Response* to treatment/day/ P.Value 0.005	Follow up / yr.	Course of disease/yr
1	M	3	uilateral	3–6	4–5	1–2	14	5
2	M	7	uilateral	2–4	3–5	1–2	20	3
3	F	4	bilateral	3–5	4–7	1–2	16	5
4	F	4	mixed	5–7	3–5	1–2	10	4
5	M	5	uilateral	3–5	4–7	1–2	8	4
6	M	6	uilateral	4–5	5–7	1–2	5	3
7	M	4	mixed	3–6	4–6	1–2	3	4
8	F	8	uilateral	5–6	4–6	1–2	12	3
9	M	5	uilateral	3–5	3–6	1–2	18	4
10	M	6	bilateral	3–6	5–7	1–2	5	3

*Swelling, pain, and fever to be resolved

The most common clinical findings were swelling (100%), pain (100%) and fever (70%). Two patients (20%) reported bilateral symptoms, while the rest had both glands affected, although not usually at the same time. All patients (100%) were lack of symptoms after 7 days, with a median duration of 4 days.

The average reported frequency was 4 episodes per year. Only 1 (10%) patient (NO-2) was diagnosed without delay, nine (90%) were diagnosed within one to two years, the longest delay to diagnosis was 2 years. (NO-6)

In comparison with their natural course of disease all patients showed a dramatic and meaningful response to treatment on the first day of administration of prednisolone (P-Value 0.005) in each attack. They were completely free symptoms on end of second day of therapy with lack of any side effect of therapy.

For ten years (mean) follow up there was not any additional accompanying autoimmune disorder.

## Discussion

JIRP is usually a self-limiting condition, resolving by adolescence, but a small number of cases have continued into adulthood [21].

Currently JIRP is an inflammatory condition, rather than viral or bacterial infections [22].

The first episode of parotitis is often attributed to mumps, in addition to mumps, other viruses such as epstein-barr virus (EBV), parainfluenza, adenovirus, human herpes virus type 6 (HHV-6), [23] hepatitis C virus [24], and HIV [25] have been implicated.

Recently, diagnostic criteria for JIRP are suggested by Garavello et al. Inclusion criteria are age < 16 years, recurrent unilateral or bilateral swelling and at least 2 episodes during the last 6 months as well as exclusion criteria: obstructive lesions, dental malocclusion, Sjögren syndrome (SS), and IgA deficiency are proposed [26].

Wu et al. concluded that immune function was altered in patients with JIRP compared to the general population, with a reduced cellular immune response and inadequate antibody production [27].

JIRP could be considered a warning sign of potential selective IgA deficiency, IgG subclass deficiencies, gastrointestinal disorders (especially coeliac disease), autoimmune diseases and even malignant tumors [25].

Development of recurrent parotitis is a common warning sign of SS. A multicenter study in 40 pediatric patients with a diagnosis of primary SS found that the most common manifestation at onset was JIRP [15].

These findings highlight the importance of performing tests for markers of autoimmune disease in patients with JIRP.

There is association of JIRP with salivary gland disease secondary to gluten enteropathy, and cystic fibrosis.

JIRP could be a new indicator to rule out coeliac disease and routine screening for coeliac disease should be tested in these patients [25].

Two retrospectively large series report among the pediatric patients identified, 1 patient with positive test for antithyroglobulin antibodies, and 2 cases with increased levels of ANA, and in 1 level of IgG and IgM in the lower limit of normal, 1 case showed decreased levels of IgA, 2 cases had increased levels of IgE [4, 28].

The older the age of onset of JIRP, the more likely there is an underlying diagnosis of Sjogren’s disease [29, 30].

During the whole of follow up there was not any additional accompanying autoimmune disorder among our case series, and although Papadopoulou et al.as like as our study did not find any positive autoantibody test results or evidence of autoimmune disease through his research [11] however, in clinical practice, JIRP could be considered a warning sign of potential immunologic and autoimmune disorders.

The diagnosis of JIRP is often confirmed by US, however, Magnetic resonance imaging (MRI) scans can also assess parotid tissue, and newer techniques such as MR sialography are now available, but not widely used [22, 31–33].

Once the diagnosis is established, management should be aimed at ameliorating symptoms with simple analgesics [22, 33]. Some treatment methods such as intraductal injection of a sclerosing substance, parotid duct ligation, parotidectomy, and radiation were included to reduce the likelihood of recurrent episodes, however, nowadays are not used as a routine modality for the treatment of JIRP [22].

Philippe Katz and colleagues used a less invasive treatment method by performing sialography and installing iodinated oil where recurrences were lesser in such treated cases [34].

Nahlieli et al. performed duct probing with lavage, dilation, and hydrocortisone injection via sialendoscopy with resolution of symptoms and a very low recurrence rate on follow-up [2].

Sialendoscopy is a newer modality in the diagnosis of salivary gland diseases. It irrigates and dilate Stensen’s duct under direct vision and provide a diagnostic and simultaneous therapeutic manner [22, 35]

The limitation of the procedure is its invasive nature and the need of anesthesia. Complications regarding this procedure include ductal perforation, anesthetic complications, and postoperative ductal stenosis [36].

Utility of steroid as a short-term and single day therapy is a known and mainstay policy of treatment in pediatric rheumatology. Periodic fever, aphthous stomatitis, pharyngitis, and adenitis (PFAPA) syndrome is a prototype of this kind of treatment in autoimmune/auto-inflammatory disorders, [37] and someway it impressed us to conduct and introduce this therapy as a non-invasive method in JIRP.

Determining some limitations of this study will help researchers in re-evaluating of our results. Small group of patients was one of them, and didn’t let us organize our study in two separate groups, case and control. Because of the rarity of this condition a multi-center study could resolve this problem.

On the other hand, new introduced therapy, Sialendoscopy was not available at our hospital, and we could not compare our results with this modality.

## Conclusion and recommendation

Although short course prednisolone on first day of episode (0.5 mg /kg) and its dramatic and meaningful response in our patients, introduce a new, effective, fast, and inexpensive regimen of therapy in patients with JIRP, however recurrent parotitis is a condition that has still some issues to be solved. More randomized trials are needed to determine the best method to treat JIRP.

### Electronic supplementary material

Below is the link to the electronic supplementary material.

Supplementary Material 1

## Abbreviations

JIRP: Juvenile idiopathic recurrent parotitis
SS: Sj ögren syndrome
US: ultrasonography
MRI: magnetic resonance imaging
EBV: Epstein-Barr virus
HHV-6: human herpes virus type 6
ANA: antinuclear antibodies
ENA: extractable nuclear antigen
CMV: Cytomegal virus
HSP: Herpes simplex Virus
VZV: Varicella Zoster virus
HIV: Human Immune deficiency virus
PFAPA: Periodic fever, aphthous stomatitis, pharyngitis, and adenitis
