# Supplementary material for: Juvenile idiopathic recurrent parotitis (JIRP) treated with short course steroids, a case series study and one decade follow up for potential autoimmune disorder
Source: Pediatr Rheumatol Online J. 2024 Jan 4;22:8. doi: 10.1186/s12969-023-00946-0 (PMC10765850; doi:10.1186/s12969-023-00946-0)
Supplement: Supplementary file 1 — Supplementary Material 1 [file 12969_2023_946_MOESM1_ESM.docx]

Dear Editor:

Regarding our article as following ID, we would like to inform you that based on reviewers’ comment- none of them rejected our article-we revised the manuscript and answered line by line to all points.

Based on mentioned and following changes would you please **re-evaluate** decision of our manuscript.

PROJ-D-23-00158

Juvenile Idiopathic Recurrent Parotitis (JIRP) treated with short course steroids, a case series study and one decade follow up for potential autoimmune disorder

Farhad Salehzadeh; Rasol Molatefi; Ali Mardi; Negin Nahanmoghaddam

Pediatric Rheumatology

Reviewer reports:

Reviewer #1: This is a case-series report of idiopathic recurrent parotitis, with intervention of short course of oral prednisolone. There are methodological issues to address and the following gaps in the data reported:

1-    The study was conducted 20 years past and the current report based on notes review. The results are very limited due to:

a. The differential diagnosis of parotid swelling is missing. Mumps, HIV, primary immunodeficiency tests were not reported.

The reviewer didn’t have careful attention to methods we explain all with details.

b. It is stated that the conservative treatment is the standard of care and treatment algorithm is based on exclusion of other diseases including Sjogren's Disease. There is lack of information on how autoimmunity was ruled out.

The reviewer didn’t have careful attention to methods we explain all with details and highlighted some of them.

c. Comparison with the natural disease course: the authors reported only one arm of the comparison, those receiving oral prednisolone and the end point was the parotid swelling reduction and symptom control (pain, fever)

We think the reviewer has misunderstanding about the natural course of the disease, natural course is the duration of each episodes alongside with frequency and prolongation of disease.

d. What was the comparison with the natural disease course, the second arm of the comparison is missing.  And how was it tested? There is a reported statistical comparison and a p-value (Table 1).

We think the reviewer has misunderstanding about the natural course of the disease, natural course is the duration of each episodes alongside with frequency and prolongation of disease

e.    The conclusion of effectiveness of prednisolone treatment is not supported by the results.

This is incorrect, looking at the table 1 and results could show its effectiveness.

Reviewer #2: This is an interesting report of the response of 10 children with Juvenile idiopathic recurrent parotitis to relatively low dose corticosteroid treatment, which rapidly  controlled symptoms, although did not alter the  overall disease course. There is no control group in this case study. In variable but long-term follow up there was no development of autoimmune disease as judged by persistently negative tests for autoantibodies. The relative simplicity, economy, and safety of this non-invasive approach to management is encouraging.

The manuscript would benefit by summarizing the discussion, shortening it by about 50%. Perhaps, noting the invasiveness of some of the tests, and the rarity of associations of JIRP with other diseases. There are several instances of unusual sentence construction or use of the English language which could be improved.

We revised it by native language.

Reviewer #3: Dear authors, after an extensive and careful assessment of your manuscript, I consider your conclusions out of reach by your data and analysis; therefore, I recommend not to be accepted as it is.

The manuscript requires a profound language edition.

We revised it by native language.

References need revision is 9-11 instead of 9, 10, and 11 (page 5,  line 33) or in line 45, the references 3, 10, 5, 12-17 must be 3, 5, 10, 12-17; please verify all of them.

We corrected all of them.

Page 7, line 15 is Wilcoxon, not Wilcoxan, and it is the alternative test for paired samples with no normal distribution.

We corrected all of them.

However, I have several questions. How did the authors reach the goal of a dramatic and meaningful response? How did they measure this response? How do we interpret the P value shown; what was the test used?

We explained the dramatic response by elimination of major symptoms, and on subtitle of the table 1 and inside the methods we have more details.

How do the authors know the subjects did not develop other diseases, where is the data?

And for what comparisons did the authors use the Wilcoxon test?

We explained 2 times about the our control group, and we compared the patients’ natural feature without treatment and after therapy by prednisolone. I wounder why reviewer did not have attention to them.

Reviewer #4: The authors of the article present a series of 7 cases of children with Juvenile Idiopathic Recurrent Parotitis (JIRP) with a 10-year follow-up for autoimmune diseases. They suggest treatment with a short course of steroids on the first day of symptoms with good clinical effect.

Comments:

1. Due to the small size of the group and the lack of comparison of the course of the disease with the group without steroids, this method of treatment cannot be considered as a strong clinical recommendation.

We explained this in methods and inside the discussion of manuscript, as limitation of study.

2. Due to the topic, the article should be sent to a general paediatric or otolaryngological journal.

Recurrent parotitis in children is the first presentation of Sjogren disease, so pediatric journal of rheumatology is the best choice.

Reviewer #5: The study that I responded to for a long term in progress after the treatment of JIRP is interesting.

However, the details of the result of a diagnosis and an image and the labo date of JIRP do not have a mention in result.

We thought negative results of our blood working are not necessary to be in results section.

Because there is little number of cases, it is thought that one information is important.

We explained this in methods and inside the discussion of manuscript, as limitation of study.

The detailed data 20 years ago must be left,

We did it.

Using the detailed data only for remaining cases, how about submit a manuscript it on case repor?

This is case series not case report.

Reviewer #6: The authors describe a group of children with juvenile idiopathic recurrent parotitis and present the results of treatment with glucocorticoids in this disease.

The manuscript needs linguistic revision.

We did it.

The following are my comments:

Abstract:

Background, line 47: delete "determined to”. We did it.

Methods, line 55: delete "We conducted this study twenty years ago, however"

We did it.

Methods, line 58: delete "usual", and "some possible background" - please specify the differential diagnoses you excluded

We did it.

Results, line 12: delete "the course of disease were 3.8 years" - this is unclear, you can specify what you mean by this in the main manuscript text.

We explained it.

Results, line 12/13: delete "The average follow up of patients was near 10 years." because you give this information also in the last sentence of the results section.

We did it.

Conclusion: delete "somehow safe", as no data on the safety of the therapy are reported.

We did it.

Main manuscript

Methods:

Line 23: delete "We conducted this study twenty years ago, and "

We did it.

Results:

Line 30: Please report the exact number of patients in the first sentence of this paragraph.

We did it.

Line 31: What about "the course of disease were 3.8 years"? Does this mean that the symptoms of recurrent parotitis occurred repeatedly during this time? Please specify.

We did it.

Line 39: "free of symptoms"

We did it.

Line 50/51: "They were completely free of symptoms at the end of the second day of therapy, although, it could not change the course of the disease".

We did it.

What does this mean? If the patient was completely free of symptoms after 2 days, what else characterized the course of the disease, which is given as 3 to 7 days in the table?

We explained it.

Were there any side effects of the therapy? Were these systematically recorded?

We explained it.

Line 53/54: "there was not any additional accompanying autoimmune disorder" - were clinical and serological examinations carried out regularly? If yes, which ones?

We explained it.

Discussion:

Line 49-54: Delete this paragraph, since the given information is redundant to the paragraph of the lines 35-38

We did it.

Conclusion, line 41: delete "somehow safe"

We did it.
